# Urban land-use filtering favors herbaceous spontaneous flora and reshapes alien-native composition in a semi-humid inland city

**DOI:** 10.3389/fpls.2026.1926163

**Published:** 2026-09-17

**Authors:** Yichao Wang, Qian Wang, Xinyi Zhang, Xinna Li, Mir Muhammad Nizamani, Qinghui Sun

**Affiliations:** 1 National Health Commission Key Laboratory of Control of Tropical Diseases, School of Life Sciences and Medical Technology, Hainan Medical University, Haikou, Hainan, China; 2 School of Environment and Geography, Qingdao University, Qingdao, China; 3 Guangdong Provincial Key Laboratory of Marine Disaster Prediction and Prevention, Institute of Marine Sciences, Shantou University, Shantou, China

**Keywords:** alien-native composition, herbaceous flora, land-use filtering, semi-humid inland city, urban functional units, urban plant diversity, urban spontaneous plants

## Abstract

Urban spontaneous plants record how vegetation responds to land use, disturbance, management, and propagule pressure, but their composition and assembly remain insufficiently studied in semi-humid inland cities. We surveyed 151 urban functional units (UFUs) in Jinan, China, combining field vegetation inventories, high-resolution land-cover interpretation, socioeconomic and management variables, and count-data robustness models to test how urban habitat filters structure spontaneous plant richness and composition. Across all UFUs, 379 vascular plant species were recorded, including 140 spontaneous species. The spontaneous flora was almost entirely herbaceous (138 species, 98.6%), with only two woody species occurring as seedlings and no mature spontaneous trees recorded. Alien species accounted for 74 spontaneous species (52.9%), slightly exceeding native species. Spontaneous richness differed among UFUs, with residential districts supporting the highest mean richness and transportation areas supporting the lowest. Exploratory linear regression identified positive associations of richness with residential land use, fertilization frequency, housing price, and UFU area. After accounting for overdispersed count data, however, negative binomial models retained residential land use and total UFU area as the most stable positive predictors, whereas fertilization and housing price were not significant. These results indicate that spontaneous plant diversity in Jinan is primarily organized by land-use filtering and habitat capacity, while socioeconomic and management gradients provide weaker and model-dependent signals. The study highlights the value of UFU-based analysis for linking urban plant community assembly with practical land-management units in rapidly urbanizing inland cities.

## 1 Introduction

Spontaneous species are plant species that establish, regenerate, and persist at a site without deliberate planting or cultivation. When such species occur within urban environments, we refer to them as urban spontaneous species (or urban spontaneous plants); the distinction is therefore one of environmental context rather than a separate biological category. Hereafter, “spontaneous plants” or “spontaneous species” refers to the urban spontaneous flora surveyed in Jinan unless otherwise stated. They occur in road verges, vacant lots, residential green spaces, park edges, wall bases, construction margins, and other microsites where urban disturbance and propagule arrival intersect. Although such plants are often removed as weeds, they are increasingly recognized as a dynamic component of urban biodiversity because they can provide pollinator resources, stabilize exposed soil, occupy small habitat patches unsuitable for planted vegetation, and create opportunities for environmental education (Del Tredici, 2010; Kowarik, 2011; Robinson and Lundholm, 2012; Gao et al., 2021). Their presence also provides a direct ecological signal of how plants respond to impervious land cover, mowing, trampling, nutrient inputs, soil disturbance, irrigation, and propagule pressure, and can reveal adaptive responses to recurrent urban disturbance (Fukano et al., 2020).

This signal is especially useful because spontaneous plants are not deliberately selected by urban planners. Unlike cultivated flora, which often reflects aesthetic preference, nursery availability, and planting design, spontaneous flora is closer to a realized outcome of colonization, establishment, disturbance, and removal (Kowarik, 2011; Hu et al., 2022). It can therefore reveal whether urban green spaces merely contain planted vegetation or also permit autonomous ecological processes. For functional plant ecology, this distinction is important because regeneration status, life form, and origin composition together indicate how urban habitats filter plant strategies rather than only species numbers.

Urban plant communities are assembled through interactions between regional species pools and local filters. In cities, land use is a particularly strong anthropogenic filter because it determines habitat amount, configuration, accessibility, substrate condition, and maintenance regime (Aronson et al., 2014; Gong et al., 2020; Qian S. et al., 2020). Administrative districts are often too coarse to capture these mechanisms. Urban functional units (UFUs), such as residential districts, transportation corridors, public service areas, commercial spaces, recreation areas, and undeveloped land, provide a finer framework because they integrate human activity, management objectives, built-up intensity, and green-space structure within spatially explicit parcels (Qian Y. et al., 2020; Zhang et al., 2023). Different UFUs therefore impose distinct habitat filters: differences in the extent of impervious land cover, substrate conditions, vegetation structure, disturbance frequency, maintenance intensity, and propagule input alter the availability of establishment microsites and the likelihood that spontaneous plants can colonize, reproduce, and persist. We consequently expected spontaneous plant richness and composition to differ among UFU types.

Socioeconomic gradients can further modify these land-use filters. The luxury effect hypothesis predicts that wealthier areas often support higher plant diversity because of greater investment in green-space construction, irrigation, fertilization, and maintenance (Hope et al., 2003; Kinzig et al., 2005; Clarke et al., 2013; Wang et al., 2015). For spontaneous flora, however, greater investment does not necessarily translate into greater richness. Intensive mowing, weeding, seedling removal, and replacement of heterogeneous ground layers with uniform lawns can suppress spontaneous recruitment even where irrigation and fertilization inputs are high. Thus, socioeconomic investment can enhance cultivated diversity while exerting neutral or negative effects on spontaneous flora. Conversely, moderate management and structurally heterogeneous residential green spaces can retain microsites where ruderal herbs, native generalists, and alien species coexist (Cheng et al., 2022; Gao et al., 2021). Separating robust land-use signals from weaker socioeconomic or management associations is therefore essential for interpreting spontaneous plant diversity.

Most Chinese studies of urban spontaneous or total plant diversity have focused on tropical coastal cities, mountainous cities, urban parks, river corridors, or large metropolitan regions (Cheng et al., 2022; Hu et al., 2022; Nizamani et al., 2023; Guo et al., 2024; Xu et al., 2024; Zhang et al., 2024; Yang et al., 2023; Chen and Wang, 2025). Semi-humid inland cities remain underrepresented, despite rapid urban expansion, strong seasonality, and intensive land conversion. Jinan, a warm-temperate semi-humid inland city in northern China, provides a suitable system for testing whether UFU type and socioeconomic context filter spontaneous plant communities under climatic conditions different from tropical urban systems. We used 151 UFUs in Jinan to ask: (i) whether spontaneous flora is dominated by herbaceous and disturbance-tolerant species, (ii) whether richness differs among UFU types, and (iii) whether socioeconomic and management variables remain robust predictors after accounting for the count-data structure of richness.

## 2 Materials and methods

### 2.1 Study area and UFU sampling

The study was conducted in Jinan, Shandong Province, eastern China (36.02-37.54°N, 116.21-117.93°E) (**Figures 1A, B**). The city lies in a warm-temperate semi-humid continental monsoon climate zone, with a mean annual temperature of approximately 14.7 °C, mean annual precipitation of approximately 671.1 mm, and pronounced seasonal variation. We focused on the main urban area and rapidly urbanizing surroundings, covering approximately 768 km2. This area contains dense residential neighborhoods, public service campuses, commercial and industrial spaces, transportation corridors, parks, and undeveloped or temporarily disturbed land, making it suitable for testing UFU-based habitat filtering.

The study area was overlaid with a 1 km x 1 km grid using high-resolution Google Earth imagery. The grid was used as a spatial guide to distribute sampling across the urbanized area rather than as a requirement that every grid cell contain a surveyed UFU; consequently, some cells in **Figure 1B** contain no sampled UFU. Candidate UFUs were selected from accessible locations within this grid framework, and UFU boundaries and land-use types were identified by manual visual interpretation and field verification in ArcGIS. Primary UFUs included public affairs service districts, industry and business districts, residential districts, recreation and leisure districts, transportation areas, and undeveloped land. Secondary UFUs included colleges or universities, governmental agencies, hospitals, research institutes, high- and low-density residential areas, parks, shopping malls, hotels, factories, roads, stations, and bareland. Primary UFUs represented broad functional land-use classes and were used for the principal comparisons, whereas secondary UFUs retained finer functional distinctions within those classes. This hierarchical classification allowed broad land-use filtering to be evaluated while preserving ecologically and managerially relevant fine-scale heterogeneity; because several secondary categories had small sample sizes, secondary-UFU results were treated mainly as supporting evidence.

**Figure 1:**
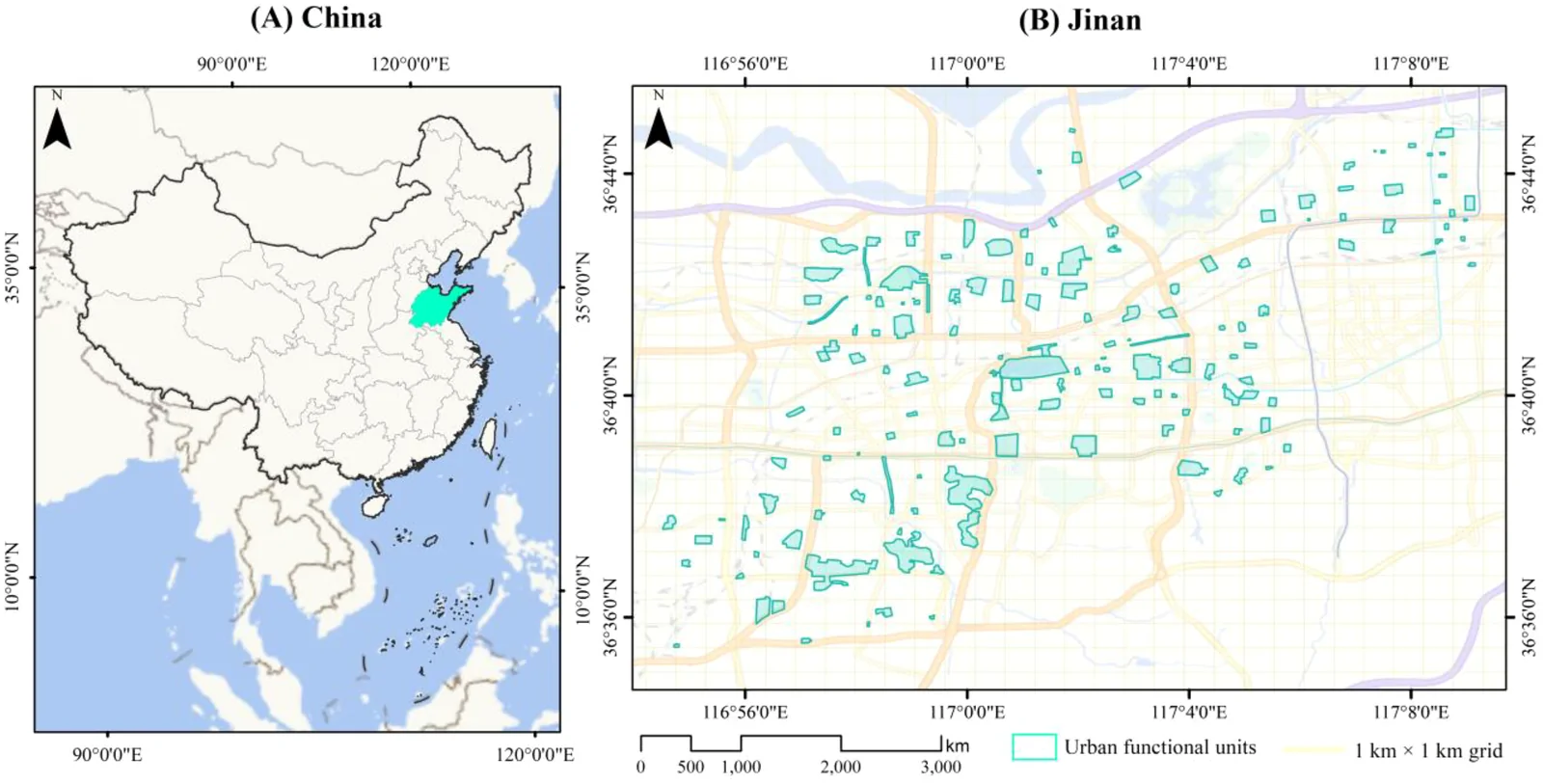
Geographic location of the study area and sampled urban functional units (UFUs) in Jinan, China. **(A)** Location of Shandong Province in China; **(B)** vector basemap of the urban study area showing the surveyed UFUs and the 1 km x 1 km sampling grid. The base map was provided by the National Geospatial Information Public Service Platform (Tianditu), with visual modifications applied by the authors.

### 2.2 Vegetation survey and species classification

Field surveys were conducted from June to October 2024. For UFUs with sufficient contiguous vegetated area, a nested quadrat design was used. A 20 m x 20 m tree quadrat was established as the base layer; within it, five 5 m x 5 m shrub quadrats were arranged at the four corners and center, and one 1 m x 1 m herb quadrat was nested within each shrub quadrat. This nested approach is consistent with multi-scale vegetation survey principles used to assess urban forest structure and plant community composition (Stohlgren et al., 1995; Nowak et al., 2008). Where a UFU did not contain sufficient contiguous vegetated area to accommodate the full 20 m x 20 m nested layout, vegetation was surveyed within the available vegetated portion using the Split Modified-Whittaker approach as a supplementary method (Stohlgren et al., 1995). Under both approaches, trees, shrubs, and herbs were recorded separately to compile a UFU-level species inventory. Because the primary response was UFU-level species richness based on species presence rather than standardized quadrat-level abundance or cover, the two field layouts were used to obtain comparable presence-based inventories across differently sized and configured urban parcels.

For each species, scientific name, growth form, phenological stage, growth condition, and growth origin were recorded. Plant identification during the field survey was assisted by the Xingse mobile application. For species that could not be reliably identified using the application, or for which identification remained uncertain, diagnostic morphological characteristics were further checked against the Flora of Shandong (Chen et al., 1990, 1997) and Flora Reipublicae Popularis Sinicae (Editorial Committee of Flora Reipublicae Popularis Sinicae, 1959-2004). These floristic references were also followed for the verification and standardization of plant names used in the species inventory. Growth origin was classified as cultivated or spontaneous according to field evidence and local planting context. Spontaneous species were defined as species not deliberately planted or maintained at the surveyed microsite and capable of colonizing, persisting, or regenerating without direct human planting. Geographic origin was classified as native or alien, and life form was classified as tree, shrub, or herb. Functional-use attributes, including edible, medicinal, and ornamental value, were compiled from published floras, local knowledge, and relevant plant-use databases; these categories were not mutually exclusive (**Figure 2**).

**Figure 2:**
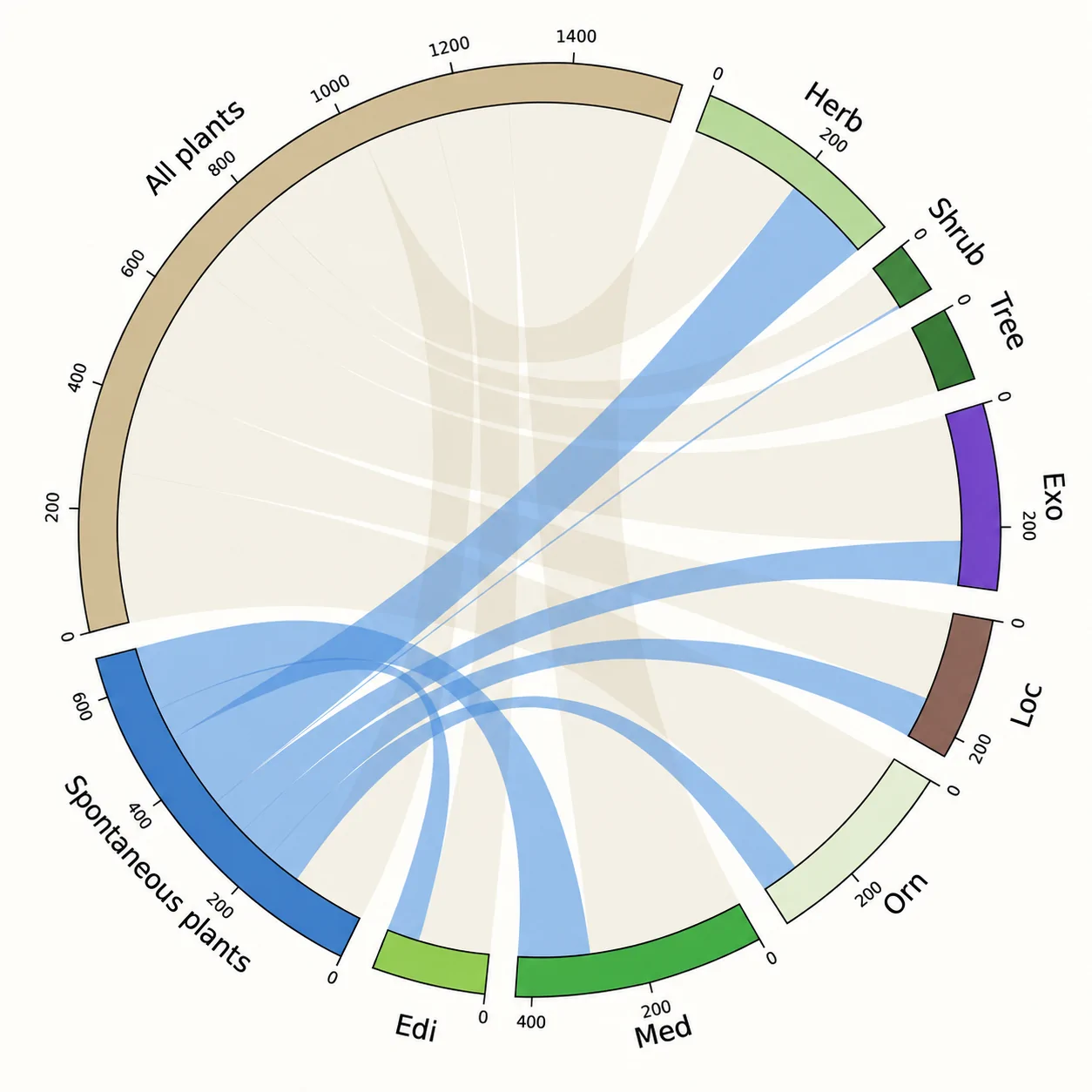
Composition of spontaneous and total plant species in Jinan. The diagram summarizes the relationships among plant status, functional-use attributes, geographic origin, and life form of spontaneous plants. Edi, edible; Med, medicinal; Orn, ornamental; Loc, local/native; Exo, exotic/alien.

The same species-identification and classification rules were applied across all UFUs to maintain comparability among functional spaces. Because the aim was to characterize spontaneous flora at the UFU scale, species presence was emphasized more than fine-scale abundance. This common presence-based endpoint allowed species-richness comparisons across UFUs surveyed with either field layout, but the results should not be interpreted as standardized estimates of plant cover, biomass, or demographic performance. Observations of woody individuals were recorded carefully because the presence or absence of mature spontaneous woody plants provides direct evidence about regeneration constraints within highly managed urban parcels.

### 2.3 Urban variables and statistical analysis

Cloud-free high-resolution Google Earth imagery from May to June 2024 was used to interpret land cover within each UFU. Land-cover interpretation and parcel-level functional classification were used to link vegetation observations with spatially explicit urban land-use structure (Gong et al., 2020; Qian Y. et al., 2020; Zhang et al., 2023). Land cover was classified into built-up area, tree and shrub area, herbaceous area, water body, and bareland. Built-up area included buildings, construction sites, paved spaces, and other forms of impervious land cover; tree and shrub area included woody vegetation patches and park woodland; herbaceous area included lawns, meadows, and low-stature herbaceous cover; and bareland referred to vacant or temporarily disturbed surfaces with low vegetation cover. These categories were used to represent habitat availability and substrate continuity within each functional parcel.

Housing price, construction age, population density, traffic flow, and distance to the nearest main or secondary road were used as socioeconomic and anthropogenic variables. Housing price was obtained from the Anjuke real estate platform, with missing values substituted using adjacent or comparable neighborhoods. Construction age was cross-validated using field interviews, building management information, and publicly available online records. Traffic flow was treated as a coarse proxy for local traffic-related disturbance, and green-space management variables included annual maintenance frequency, fertilization frequency, and watering frequency. Environmental variables included mean annual temperature, wind speed, and solar radiation. Because climatic variation within the urban area was limited relative to land-use and management variation, these variables were interpreted as background environmental covariates rather than primary experimental treatments.

Spontaneous plant richness, defined as the number of spontaneous species recorded in each UFU, was the primary response variable. Differences in richness among UFU categories were evaluated using the Kruskal-Wallis rank-sum test followed by pairwise Wilcoxon rank-sum tests with Benjamini-Hochberg adjustment. Continuous variables were standardized using z-scores before regression analysis, and extreme outliers with absolute z-scores greater than 3 were excluded from model fitting. Exploratory multiple linear regression models were first fitted to identify associations between richness and UFU type, land-cover variables, socioeconomic variables, management variables, traffic variables, climate covariates, and total UFU area. Because richness is non-negative count data and residual distributions deviated from normality, generalized linear models were also fitted. A Poisson generalized linear model with a log link was first used, overdispersion was assessed using the Pearson residual dispersion ratio, and a negative binomial model was fitted when overdispersion was detected. Incidence rate ratios (IRRs) and 95% confidence intervals were calculated by exponentiating model coefficients. Analyses were conducted in R 4.4.1 and Python-based statistical workflows. Data visualization was performed using the ggplot2 package (Wickham, 2016).

## 3 Results

### 3.1 Spontaneous flora was herbaceous and alien-rich

Across the 151 surveyed UFUs, 379 vascular plant species were recorded, of which 140 were classified as spontaneous (**Supplementary Tables 1**, **S5**). The spontaneous flora was strongly dominated by herbs: 138 spontaneous species were herbaceous, representing 98.6% of all spontaneous species, whereas only two woody species were recorded as seedlings. No mature spontaneous tree individuals were observed. Frequently recorded spontaneous species included *Digitaria sanguinalis* (L.) Scop., *Setaria viridis* (L.) P. Beauv., *Ophiopogon bodinieri* H.Lév., *Eleusine indica* (L.) Gaertn., *Portulaca oleracea* L., *Chenopodium album* L., *Oxalis corniculata* L., *Alternanthera philoxeroides* (Mart.) Griseb., *Amaranthus retroflexus* L., and *Sonchus oleraceus* L. A complete list of the spontaneous species recorded is provided in **Supplementary Table 5**.

Alien species slightly outnumbered native species within the spontaneous flora. Native species accounted for 66 species (47.1%), whereas alien species accounted for 74 species (52.9%). Functional-use attributes were common: 55 spontaneous species had edible value, 121 had medicinal value, and 60 had ornamental value. These results indicate that spontaneous flora in Jinan is characterized by herbaceous dominance, limited woody regeneration, a substantial alien component, and broad functional-use potential (**Figure 2**; **Table 1**; **Supplementary Table 1**).

**Table 1:** Condensed empirical and count-data results for spontaneous plants in Jinan.

Component	Result
Survey scope	151 urban functional units; 379 vascular plant species recorded.
Spontaneous flora	140 spontaneous species; 138 herbaceous species (98.6%); 2 woody seedlings; no mature spontaneous tree species.
Geographic origin	66 native species (47.1%) and 74 alien species (52.9%).
Functional-use attributes	55 edible, 121 medicinal, and 60 ornamental spontaneous species; categories are not mutually exclusive.
Primary UFU richness	Residential districts: 11.00 ± 3.12; undeveloped land: 9.25 ± 5.67; recreation/leisure: 8.33 ± 4.44; public affairs service: 6.88 ± 4.30; industry/business: 6.39 ± 7.48; transportation: 4.43 ± 4.10.
Exploratory linear model	Residential districts: standardized beta = 1.297, p < 0.001; fertilization frequency: beta = 0.262, p = 0.003; housing price: beta = 0.191, p = 0.035; total UFU area: beta = 0.197, p = 0.049.
Count-data model fit	Primary Poisson GLM: AIC = 556.086, dispersion = 2.945; primary negative binomial GLM: AIC = 519.641.
Robust predictors in NB model	Residential districts: IRR = 1.882, 95% CI = 1.237-2.863, p = 0.003; total UFU area: IRR = 1.241, 95% CI = 1.042-1.479, p = 0.016; wind speed: IRR = 0.770, 95% CI = 0.625-0.948, p = 0.014.
Model-dependent predictors	Fertilization frequency and housing price were positive in exploratory linear models but not significant in the negative binomial model.

### 3.2 UFU type differentiated spontaneous richness

Spontaneous plant richness differed among primary UFUs, with residential districts showing the highest mean richness and transportation areas the lowest (**Figure 3A**; **Supplementary Table 2**).

**Figure 3:**
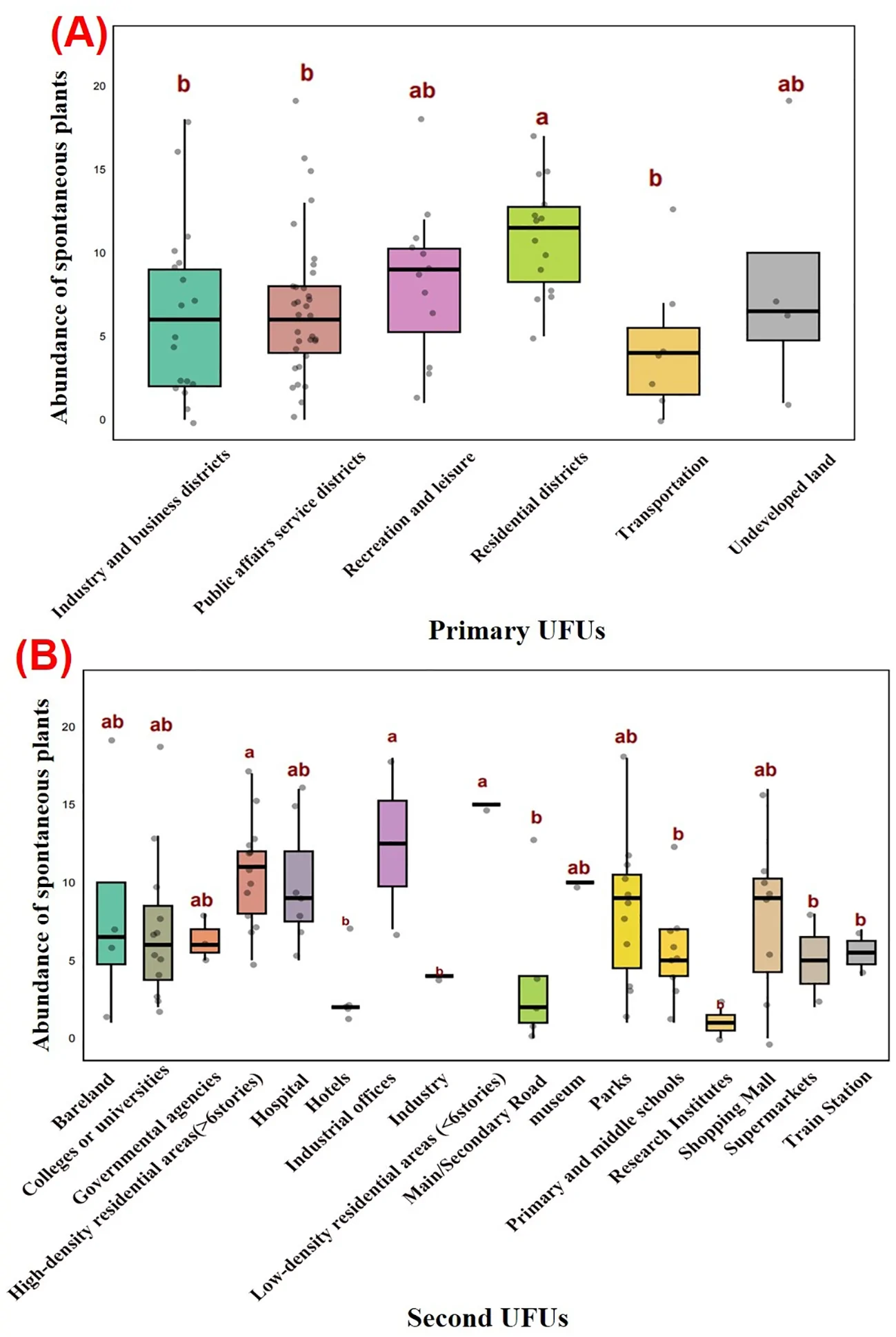
Differences in spontaneous plant richness among UFU types. **(A)** Primary UFUs; **(B)** secondary UFUs. Boxes show interquartile ranges, horizontal lines show medians, points indicate observations, and letters indicate significant pairwise differences after multiple-comparison adjustment.

Secondary UFUs also showed fine-scale variation in richness (**Figure 3B**). Because several secondary categories had small sample sizes, these patterns were treated as descriptive supporting evidence rather than the main inferential basis.

### 3.3 Count-data models identified robust predictors

Exploratory regression supported a UFU-based filtering interpretation. In the primary-UFU linear model, using industry and business districts as the reference category, residential districts showed a strong positive association with spontaneous richness (standardized beta = 1.297, p < 0.001). Fertilization frequency (beta = 0.262, p = 0.003), housing price (beta = 0.191, p = 0.035), and total UFU area (beta = 0.197, p = 0.049) were also positively associated with richness. Maintenance frequency, population density, construction age, traffic flow, road distance, and climate covariates were not significant in this model (**Supplementary Table 3**).

The primary Poisson model showed clear overdispersion, with a Pearson dispersion ratio of 2.945. The negative binomial model had a lower AIC than the Poisson model (519.641 versus 556.086), indicating a better fit to the count-based richness data. In the negative binomial model, residential districts remained significantly and positively associated with richness relative to industry and business districts (IRR = 1.882, 95% CI = 1.237-2.863, p = 0.003). Total UFU area was also a significant positive predictor (IRR = 1.241, 95% CI = 1.042-1.479, p = 0.016), whereas wind speed showed a negative association (IRR = 0.770, 95% CI = 0.625-0.948, p = 0.014). Fertilization frequency and housing price, although positive in direction, were not significant after accounting for overdispersion. Thus, residential land use and habitat area were the most stable predictors of spontaneous richness (**Table 1**; **Supplementary Table 4**; **Supplementary Figure 2**).

## 4 Discussion

This study shows that spontaneous plant communities in Jinan are strongly structured by urban land-use filtering. The flora was overwhelmingly herbaceous, contained slightly more alien than native species, and showed almost no woody regeneration beyond scattered seedlings. These patterns support the expectation that frequent mowing, cleaning, trampling, soil disturbance, and surface hardening restrict long establishment windows required by woody plants, while favoring short-lived or disturbance-tolerant herbs (Wang et al., 2015; Hu et al., 2022; Chen and Wang, 2025). Species such as *Digitaria sanguinalis*, *Setaria viridis*, *Eleusine indica*, and *Portulaca oleracea* can complete life cycles rapidly, exploit exposed or compacted soils, and recolonize after disturbance. Such trait-mediated persistence is consistent with evidence that urban and rural populations can diverge in competitive and disturbance-related traits (Fukano et al., 2020). Spontaneous flora can therefore be interpreted as a sensitive indicator of recurrent urban disturbance and microsite availability.

The absence of mature spontaneous trees in the surveyed Jinan UFUs contrasts with evidence from European cities, where spontaneous woody recruitment and escape from cultivation are well documented. In Berlin, spontaneous woody flora included both native and alien trees, shrubs, and vines; among 181 alien woody species recorded in the city flora, 32% were naturalized, and alien woody richness was particularly high in green spaces, wastelands, and residential areas (Kowarik et al., 2013). In Radolfzell, Germany, Mayer et al. (2017) recorded 1,268 ornamental garden-plant species, including 954 aliens, of which 48 had already been recorded in the wild locally, illustrating how public and private plantings can provide propagules for naturalization. Similarly, the Czech survey by Pergl et al. (2016) identified ornamental horticulture as an important source of escaped and persistent wild-growing flora. By contrast, the naturalized and invasive flora documented in rapidly urbanizing Beijing was dominated largely by annual herbs associated with disturbed sites, and districts experiencing stronger recent urban and economic growth contained more naturalized and invasive species (Wang et al., 2011). Against this comparative background, the lack of mature spontaneous trees in our Jinan survey is consistent with a strong post-establishment filter: woody propagules or seedlings can occur, but repeated mowing, weeding, seedling removal, surface hardening, and landscape maintenance can shorten the persistence window needed for individuals to reach maturity. However, our study did not quantify management intensity using a standardized cross-city comparison, so we cannot conclude that Jinan is more intensively managed than the European cities cited above; management is therefore interpreted as a plausible local mechanism rather than a demonstrated cross-regional cause.

The alien-rich composition of the spontaneous flora also reflects urban disturbance and propagule pressure. Cities promote alien species through horticultural introductions, transportation networks, disturbed soils, and frequent human-mediated dispersal (Aronson et al., 2014; Kowarik, 2011; Hu et al., 2022; Li et al., 2023; Zhang et al., 2024). In Jinan, alien species slightly exceeded native species, but this does not imply that all alien taxa are invasive. Rather, it indicates that urban habitats provide frequent establishment opportunities for species with broad ecological tolerance and multiple dispersal pathways. Native species remained important in residential green spaces, park margins, and less intensively managed microsites, suggesting that spontaneous vegetation can include both conservation-relevant native plants and alien taxa requiring risk-based monitoring.

The UFU framework identified residential districts as the most consistently species-rich functional spaces. During our field surveys, spontaneous plants in higher-housing-price residential UFUs were repeatedly observed in small microsites such as gaps along curbstones and within lawns. These direct field observations show that spontaneous vegetation can persist within otherwise well-maintained residential landscapes where small establishment sites remain available. More generally, residential areas can combine heterogeneous planting beds, edge habitats, variable maintenance, and frequent propagule arrival, creating multiple microsites for herbaceous colonization and coexistence (Wang et al., 2015; Zhang et al., 2023). By contrast, transportation areas, research institutes, and some commercial spaces showed lower richness, probably because of high proportions of impervious land cover, repeated clearing, compacted substrates, limited soil volume, or strong enclosure management. The robust positive effect of total UFU area further supports the importance of habitat capacity. Larger UFUs are more likely to include diverse microsites, greater edge length, variable management intensity, and multiple dispersal opportunities, all of which can increase spontaneous richness (Gao et al., 2021; Cheng et al., 2022; Xu et al., 2024; Yang et al., 2023). These observations provide field context for the residential pattern but do not establish housing price itself as a causal driver.

The contrast between exploratory linear models and negative binomial models is important. Housing price and fertilization frequency were significant in the linear model, which is consistent with a possible luxury or greening-input signal (Hope et al., 2003; Kinzig et al., 2005; Clarke et al., 2013; Wang et al., 2015). However, these variables were not significant in the count-data model. This suggests that socioeconomic and management effects should be interpreted as secondary and context dependent rather than as stable causal mechanisms. Management inputs can have opposing effects: fertilization and irrigation can improve local growing conditions, but mowing and weeding can remove spontaneous plants before they reproduce. Aligning statistical models with the count and overdispersed structure of richness therefore reduces the risk of overinterpreting weak socioeconomic signals.

This statistical point has ecological relevance. Richness data collected from heterogeneous urban parcels are often zero-bounded, right-skewed, and overdispersed because some sites contain many more microsites than others. A predictor that appears significant in a linear model can partly reflect these distributional properties rather than a generalizable mechanism. By treating the negative binomial model as a robustness analysis rather than replacing field interpretation with a single model output, the study retains exploratory ecological breadth while emphasizing the predictors that remain stable under a more appropriate data-generating assumption.

The semi-humid inland setting should also be considered when interpreting these results. Compared with tropical coastal cities, Jinan experiences stronger seasonal temperature variation and a distinct winter period, which can shorten establishment windows for many spontaneous plants and favor annual or perennial herbs able to complete growth rapidly during suitable seasons. At the same time, urban irrigation, fertilization, and planting-bed management can create small favorable patches within an otherwise stressful urban matrix. The resulting pattern is therefore not simply a climatic signal; it reflects the interaction between regional seasonality and fine-scale urban management. This interaction could help explain why land-use function and UFU area remained more stable predictors than broad socioeconomic proxies, a contrast that complements evidence from tropical coastal cities, mountainous cities, urban parks, and urban river corridors (Cheng et al., 2022; Nizamani et al., 2023; Guo et al., 2024; Xu et al., 2024; Li et al., 2023).

These findings have practical implications for urban biodiversity management. Spontaneous herbs should not be treated uniformly as undesirable weeds; low-risk native or functionally valuable species could be retained in appropriate locations (Del Tredici, 2010; Kowarik, 2011; Robinson and Lundholm, 2012). UFU-specific management is also needed. Where spontaneous plant diversity is a management goal, transportation corridors and highly built-up spaces can benefit from soil restoration, reduced clearing frequency, and better-designed planting strips, whereas residential areas can use mosaic mowing and selective retention of non-invasive herbs. Alien spontaneous species should be evaluated according to invasion risk rather than removed indiscriminately. Such differentiated management can help balance biodiversity conservation, landscape aesthetics, public safety, and invasion-risk control.

In this sense, the UFU framework is not only a classification tool but also a management bridge. It allows plant diversity patterns to be translated into functional spaces where management actions are actually implemented. For example, the same spontaneous species can require different treatment in residential courtyards, road verges, public institutions, and undeveloped land. This practical linkage strengthens the relevance of the results for urban green-space planning and supports a shift from uniform weed removal toward risk-based and context-sensitive vegetation management (Kowarik, 2011; Robinson and Lundholm, 2012; Zhang et al., 2024).

This study has several limitations. The data are observational and cross-sectional, so the results indicate associations rather than direct causation. Some socioeconomic and management variables were derived from interviews, online platforms, or short-term field observations, which can introduce measurement uncertainty. Several secondary UFU categories had small sample sizes, limiting the strength of fine-scale inference. In addition, this study focused on species richness and broad origin categories; abundance, functional traits, phylogenetic structure, soil properties, seed banks, and temporal dynamics were not fully evaluated. Future studies should combine repeated surveys, management experiments, and trait-based analyses to distinguish colonization, persistence, and removal processes, as suggested by recent studies on microhabitats, park environments, and life-form-specific responses of spontaneous plants (Li et al., 2023; Xu et al., 2024; Yang et al., 2023; Zhang et al., 2024). Seed-bank sampling and soil measurements would be especially useful for separating propagule limitation from post-establishment filtering. Repeated surveys across seasons and years would also clarify whether the high alien proportion reflects persistent community composition or short-term pulses of ruderal recruitment after disturbance. Despite these limitations, the results show that in a semi-humid inland city, spontaneous plant communities are structured primarily by UFU-based land-use filtering and habitat capacity.

## Data Availability

The original contributions presented in the study are included in the article/**Supplementary Material**. Further inquiries can be directed to the corresponding author.

## Supplementary material

Detailed exploratory linear regression outputs, secondary-UFU model outputs, the dominant-species spatial map, count-data model coefficient plots, sampling-framework and fieldwork illustrations, and supporting diagnostics are provided as **Supplementary Material**.
