# Supplementary material for: Urban land-use filtering favors herbaceous spontaneous flora and reshapes alien-native composition in a semi-humid inland city
Source: Front Plant Sci. 2026 Sep 17;17:1926163. doi: 10.3389/fpls.2026.1926163 (PMC13627410; doi:10.3389/fpls.2026.1926163)
Supplement: Supplementary file 1 [file SupplementaryFile1.docx]

**Supplementary Material**

***Urban land-use filtering favors herbaceous spontaneous flora and reshapes alien-native composition in a semi-humid inland city***

Yichao Wang, Xinyi Zhang, Qian Wang, Xinna Li, Mir Muhammad Nizamani, Qinghui Sun

# Supplementary AI-use disclosure

Generative AI tools were used only to assist with preparation or standardization of non-data illustrative schematic elements in the Supplementary Material, including sampling-framework graphics and plant-icon elements where retained. No generative AI tools were used to generate, alter, or interpret field-observed species records, UFU boundaries, statistical outputs, model coefficients, field photographs, or empirical results. The authors reviewed and verified all AI-assisted visual elements and take full responsibility for the submitted content.

# Contents

- Supplementary Figure S1. Spatial distribution of dominant spontaneous plant species across surveyed UFUs in Jinan.
- Supplementary Figure S2. Negative binomial robustness model for spontaneous plant richness.
- Supplementary Figure S3. Hierarchical nested sampling framework and fieldwork illustrations.
- Supplementary Table S1. Summary of spontaneous plant composition in Jinan.
- Supplementary Table S2. Mean spontaneous plant richness across primary urban functional units.
- Supplementary Table S3. Exploratory primary-UFU linear regression results for spontaneous plant richness.
- Supplementary Table S4. Count-data model diagnostics and primary negative binomial results.
- Supplementary Table S5. List of spontaneous plant species recorded in Jinan.

# Supplementary figures

**Supplementary Figure S1. Spatial distribution of dominant spontaneous plant species across surveyed UFUs in Jinan.**

Plant icons indicate the predominant spontaneous species recorded in each UFU, and cyan polygons show UFU boundaries. Plant-icon elements are illustrative aids and do not replace the field-based taxonomic records used for analysis.

**Supplementary Figure S2. Negative binomial robustness model for spontaneous plant richness.**

Points indicate incidence rate ratios (IRRs), horizontal bars indicate 95% confidence intervals, and the dashed vertical line indicates IRR = 1. Values greater than 1 represent positive associations with spontaneous species richness, whereas values less than 1 represent negative associations.

**Supplementary Figure S3. Hierarchical nested sampling framework and fieldwork illustrations.**

The diagram summarizes the nested tree-shrub-herb sampling framework, including 20 m x 20 m tree plots, five 5 m x 5 m shrub plots arranged at four corners and the center, and 1 m x 1 m herb plots. Field photographs illustrate representative quadrats and maintenance activities. Schematic elements were standardized using AI-assisted graphic tools; field photographs were original and were not AI-generated or AI-altered.

# Supplementary tables

**Supplementary Table S1. Summary of spontaneous plant composition in Jinan.**

| **Category** | **Number of species** | **Percentage of spontaneous flora (%)** |
| --- | --- | --- |
| Total spontaneous species | 140 | 100.0 |
| Herbaceous species | 138 | 98.6 |
| Shrub species | 2 | 1.4 |
| Mature spontaneous tree species | 0 | 0.0 |
| Native species | 66 | 47.1 |
| Alien species | 74 | 52.9 |
| Edible species | 55 | 39.3 |
| Medicinal species | 121 | 86.4 |
| Ornamental species | 60 | 42.9 |

**Supplementary Table S2. Mean spontaneous plant richness across primary urban functional units.**

| **Primary UFU** | **Mean richness +/- SD** |
| --- | --- |
| Residential districts | 11.00 +/- 3.12 |
| Undeveloped land | 9.25 +/- 5.67 |
| Recreation and leisure districts | 8.33 +/- 4.44 |
| Public affairs service districts | 6.88 +/- 4.30 |
| Industry and business districts | 6.39 +/- 7.48 |
| Transportation | 4.43 +/- 4.10 |

**Supplementary Table S3. Exploratory primary-UFU linear regression results for spontaneous plant richness.**

| **Predictor** | **Standardized beta** | **p-value** |
| --- | --- | --- |
| Public affairs service districts | 0.301 | 0.214 |
| Recreation and leisure districts | 0.476 | 0.365 |
| Residential districts | 1.297 | <0.001 |
| Transportation | -0.116 | 0.734 |
| Undeveloped land | 0.013 | 0.974 |
| Maintenance frequency | -0.022 | 0.820 |
| Fertilization frequency | 0.262 | 0.003 |
| Green-space proportion / per-capita proxy | 0.227 | 0.141 |
| Housing price | 0.191 | 0.035 |
| Population density | -0.044 | 0.602 |
| Construction age | -0.111 | 0.195 |
| Traffic flow | -0.027 | 0.771 |
| Distance from main road | -0.065 | 0.444 |
| Mean annual temperature | -0.127 | 0.170 |
| Wind speed | -0.002 | 0.984 |
| Solar radiation | 0.010 | 0.923 |
| Total UFU area | 0.197 | 0.049 |

Industry and business districts were used as the reference category for primary UFU type. Continuous variables were standardized before model fitting.

**Supplementary Table S4. Count-data model diagnostics and primary negative binomial results.**

| **Model or predictor** | **Statistic / IRR** | **95% CI** | **p-value** |
| --- | --- | --- | --- |
| Primary Poisson GLM | AIC = 556.086; dispersion = 2.945 |  |  |
| Primary negative binomial GLM | AIC = 519.641 |  |  |
| Residential districts | IRR = 1.882 | 1.237-2.863 | 0.003 |
| Transportation | IRR = 0.628 | 0.354-1.113 | 0.111 |
| Fertilization frequency | IRR = 1.007 | 0.871-1.163 | 0.927 |
| Housing price | IRR = 1.039 | 0.898-1.202 | 0.606 |
| Wind speed | IRR = 0.770 | 0.625-0.948 | 0.014 |
| Total UFU area | IRR = 1.241 | 1.042-1.479 | 0.016 |
| Secondary Poisson GLM | AIC = 602.159; dispersion = 2.672 |  |  |
| Secondary negative binomial GLM | AIC = 571.608 |  |  |

IRR, incidence rate ratio; CI, confidence interval. The secondary-UFU model was used as supporting evidence because several secondary categories had small sample sizes.

Note. Supplementary Table S5 provides the complete list of spontaneous plant species recorded in the field survey.

# Supplementary Table S5. List of spontaneous plant species recorded in Jinan.

| **No.** | **Chinese name** | **Scientific name** | **Life form** | **Geographic origin** |
| --- | --- | --- | --- | --- |
| 1 | 银杏（幼苗） | *Ginkgo* *biloba* L., 1771 | Shrub | Native |
| 2 | 台湾栾树 | *Koelreuteria* *elegans* subsp. *formosana* (Hayata) F. G. Mey., 1976 | Shrub | Alien |
| 3 | 酢浆草 | *Oxalis* *corniculata* Linn. | Herb | Native |
| 4 | 钻叶紫菀 | *Symphyotrichum* *subulatum* (Michx.) G. L. Nesom, 1995 | Herb | Alien |
| 5 | 紫茉莉 | *Jasminum* *sambac* (Linnaeus) Aiton | Herb | Alien |
| 6 | 苎麻 | *Boehmeria* *nivea* (L.) Hook. f. & Arn. | Herb | Native |
| 7 | 竹节草 | *Chrysopogon* *aciculatus* (Retz.) Trin. | Herb | Alien |
| 8 | 猪毛草 | *Schoenoplectiella* *wallichii* (Nees) Lye, 2003 | Herb | Alien |
| 9 | 皱果苋 | *Amaranthus* *viridis* L., 1763 | Herb | Alien |
| 10 | 中华苦荬菜 | *Ixeris* *chinensis* (Thunb. ex Thunb.) Nakai | Herb | Native |
| 11 | 中华苦苣菜 | *Sonchus* *oleraceus* (L.) L. | Herb | Alien |
| 12 | 芝麻菜二行芥 | *Diplotaxis* *erucoides* (L.) DC., 1821 | Herb | Alien |
| 13 | 芝麻菜 | *Eruca* *vesicaria* subsp. *sativa* (Mill.) Thell., 1918 | Herb | Alien |
| 14 | 早开堇菜 | *Viola* *prionantha* Bunge | Herb | Native |
| 15 | 月光花 | *Ipomoea* *alba* L. | Herb | Alien |
| 16 | 圆叶牵牛 | *Ipomoea* *purpurea* (L.) Roth | Herb | Alien |
| 17 | 圆叶堇菜 | *Viola* *striatella* H. Boissieu | Herb | Alien |
| 18 | 榆树苗 | *Ulmus* *pumila* L. | Herb | Native |
| 19 | 异型莎草 | *Cyperus* *difformis* Linn. | Herb | Native |
| 20 | 一年蓬 | *Erigeron* *annuus* (L.) Desf. | Herb | Alien |
| 21 | 野莴苣 | *Lactuca* *serriola* L. | Herb | Alien |
| 22 | 野老鹳草 | *Geranium* *carolinianum* Linn. | Herb | Alien |
| 23 | 野艾蒿 | *Artemisia* *lavandulifolia* DC. | Herb | Native |
| 24 | 雪里蕻 | *Brassica* *juncea* 'Multiceps' | Herb | Native |
| 25 | 栾树苗 | *Koelreuteria* *paniculata* Laxm | Herb | Native |
| 26 | 小萱草 | *Hemerocallis* *dumortieri* Morr. | Herb | Alien |
| 27 | 小心叶薯 | *Ipomoea* *obscura* (Linn.) Ker-Gawl. | Herb | Alien |
| 28 | 瓜叶菊 | *Pericallis* *hybrida* B. Nord. | Herb | Alien |
| 29 | 小蓬草 | *Erigeron* *canadensis* L. | Herb | Alien |
| 30 | 小画眉草 | *Eragrostis* *minor* Host | Herb | Native |
| 31 | 小葱 | *Allium* *fistulosum* L., 1753 | Herb | Alien |
| 32 | 香附子 | *Cyperus* *rotundus* Linn. | Herb | Native |
| 33 | 细裂叶莲蒿 | *Artemisia* *stechmanniana* Besser, 1832 | Herb | Alien |
| 34 | 喜旱莲子草 | *Alternanthera* *philoxeroides* (Mart.) Griseb., 1879 | Herb | Alien |
| 35 | 西瓜 | *Citrullus* *lanatus* (Thunb.) Matsum. & Nakai | Herb | Native |
| 36 | 乌蔹梅 | *Causonis* *japonica* (Thunb.) Raf. | Herb | Alien |
| 37 | 土人参 | *Talinum* *paniculatum* (Jacq.) Gaertn., 1791 | Herb | Alien |
| 38 | 苘麻 | *Abutilon* *theophrasti* Medicus | Herb | Native |
| 39 | 茼蒿 | *Glebionis* *coronaria* (L.) Cass. ex Spach | Herb | Native |
| 40 | 通泉草 | *Mazus* *pumilus* (Burm. f.) Steenis, 1958 | Herb | Alien |
| 41 | 铁苋菜 | *Acalypha* *australis* Linn. | Herb | Native |
| 42 | 甜瓜 | *Cucumis* *melo* Linn. | Herb | Alien |
| 43 | 田旋花 | *Convolvulus* *arvensis* Linn. | Herb | Native |
| 44 | 天名精 | *Carpesium* *abrotanoides* Linn. | Herb | Native |
| 45 | 酸模 | *Rumex* *acetosa* Linn. | Herb | Native |
| 46 | 苏门白酒草 | *Erigeron* *sumatrensis* Retz. | Herb | Alien |
| 47 | 蛇莓 | *Duchesnea* *indica* (Andr.) Focke | Herb | Native |
| 48 | 商陆 | *Phytolacca* *acinosa* Roxb. | Herb | Alien |
| 49 | 山桃草 | *Oenothera* *lindheimeri* (Engelm. & A. Gray) W.L. Wagner & Hoch | Herb | Native |
| 50 | 三叶草 | *Oxalis* *rubra* St.-Hil. | Herb | Native |
| 51 | 软叶丝兰 | *Yucca* *flaccida* Haw. | Herb | Alien |
| 52 | 柔毛打碗花 | *Calystegia* *pubescens* Lindl. | Herb | Alien |
| 53 | 热带求米草 | *Oplismenus* *hirtellus* (L.) Beauv. | Herb | Alien |
| 54 | 茜草 | *Rubia* *cordifolia* Linn. | Herb | Alien |
| 55 | 牵牛花 | *Ipomoea* *nil* (L.) Roth | Herb | Alien |
| 56 | 七叶树 | *Aesculus* *chinensis* Bunge | Herb | Native |
| 57 | 蒲公英 | *Taraxacum* *mongolicum* Hand.-Mazz., 1907 | Herb | Native |
| 58 | 婆婆丁 | *Taraxacum* *mongolicum* Hand.-Mazz., 1907 | Herb | Native |
| 59 | 爬山虎 | *Ampelopsis* *tricuspidata* Siebold et Zucc. | Herb | Native |
| 60 | 牛膝 | *Achyranthes* *bidentata* Blume, 1825 | Herb | Native |
| 61 | 牛筋草 | *Eleusine* *indica* (Linn.) Gaertn. | Herb | Native |
| 62 | 柠檬草 | *Cymbopogon* *citratus* (DC.) Stapf | Herb | Alien |
| 63 | 茑萝松 | *Quamoclit* *pennata* (Desr.) Boj. | Herb | Alien |
| 64 | 南瓜 | *Cucurbita* *moschata* Duchesne | Herb | Alien |
| 65 | 蜜蜂花 | *Melissa* *axillaris* (Benth.) Bakh. f. | Herb | Native |
| 66 | 毛白前 | *Vincetoxicum* *chinense* S. Moore, 1875 | Herb | Alien |
| 67 | 马唐 | *Digitaria* *sanguinalis* (Linn.) Scop. | Herb | Alien |
| 68 | 马齿苋 | *Portulaca* *oleracea* Linn. | Herb | Native |
| 69 | 葎草 | *Humulus* *scandens* (Lour.) Merr. | Herb | Native |
| 70 | 绿苋草 | *Alternanthera* *ficoidea* (L.) Sm. | Herb | Alien |
| 71 | 落葵 | *Basella* *alba* L., 1753 | Herb | Native |
| 72 | 芦苇 | *Phragmites* *australis* (Cav.) Trin. ex Steud. | Herb | Native |
| 73 | 龙葵 | *Solanum* *nigrum* L., 1753 | Herb | Native |
| 74 | 蓼子草 | *Persicaria* *criopolitana* (Hance) Migo, 1939 | Herb | Native |
| 75 | 楝 | *Melia* *azedarach* Linn. | Herb | Alien |
| 76 | 莲蓬 | *Nelumbo* *nucifera* Gaertn. | Herb | Alien |
| 77 | 粟米草 | *Trigastrotheca* *stricta* (L.) Thulin, 2016 | Herb | Alien |
| 78 | 鳢肠 | *Eclipta* *prostrata* (Linn.) Linn. | Herb | Native |
| 79 | 藜 | *Chenopodium* *album* L. | Herb | Alien |
| 80 | 辣椒 | *Capsicum* *annuum* Linn. | Herb | Alien |
| 81 | 苦荬菜 | *Ixeris* *polycephala* Cass. ex DC. | Herb | Alien |
| 82 | 苦苣菜 | *Sonchus* *oleraceus* (L.) L. | Herb | Alien |
| 83 | 苦菜 | *Patrinia* *heterophylla* subsp. *angustifolia* (Hemsl.) H. J. Wang | Herb | Alien |
| 84 | 糠稷 | *Panicum* *bisulcatum* Thunb. | Herb | Alien |
| 85 | 具芒碎米莎草 | *Cyperus* *microiria* Steud. | Herb | Native |
| 86 | 菊苣 | *Cichorium* *intybus* Linn. | Herb | Alien |
| 87 | 韭菜 | *Allium* *tuberosum* Rottler ex Spreng., 1825 | Herb | Native |
| 88 | 金鸡菊 | *Coreopsis* *basalis* (A.Dietr.) S.F.Blake | Herb | Alien |
| 89 | 结缕草 | *Zoysia* *japonica* Steud. | Herb | Native |
| 90 | 绞股蓝 | *Gynostemma* *pentaphyllum* (Thunb.) Makino | Herb | Native |
| 91 | 尖裂假还阳参 | *Crepidiastrum* *sonchifolium* (Maximowicz) Pak & Kawano | Herb | Alien |
| 92 | 荠菜 | *Capsella* *bursa-pastoris* (L.) Medik., 1792 | Herb | Native |
| 93 | 鸡屎藤 | *Paederia* *foetida* L., 1767 | Herb | Alien |
| 94 | 黄花蒿 | *Artemisia* *annua* L. | Herb | Native |
| 95 | 黄鹤菜 | *Youngia* *sericea* C. Shih | Herb | Native |
| 96 | 黄菖蒲 | *Iris* *pseudacorus* Linn. | Herb | Alien |
| 97 | 黄鹌菜 | *Youngia* *japonica* (Linn.) DC. | Herb | Native |
| 98 | 画眉草 | *Eragrostis* *pilosa* (Linn.) Beauv. | Herb | Native |
| 99 | 华萝藦 | *Cynanchum* *hemsleyanum* (Oliv.) Liede & Khanum, 2016 | Herb | Alien |
| 100 | 花叶子滇苦菜 | *Sonchus* *asper* (L.) Hill, 1769 | Herb | Alien |
| 101 | 花叶苦滇菜 | *Sonchus* *asper* (L.) Hill, 1769 | Herb | Alien |
| 102 | 花叶滇苦菜 | *Sonchus* *asper* (L.) Hill, 1769 | Herb | Native |
| 103 | 虎皮菊 | *Gaillardia* *pulchella* 'Picta' A. Gray | Herb | Alien |
| 104 | 红苋菜 | *Amaranthus* *viridis* L., 1763 | Herb | Native |
| 105 | 黑桑苗 | *Morus* *nigra* Linn. | Herb | Alien |
| 106 | 何首乌 | *Pleuropterus* *multiflorus* (Thunb.) Nakai, 1914 | Herb | Native |
| 107 | 含羞草 | *Mimosa* *pudica* Linn. | Herb | Alien |
| 108 | 过江藤 | *Phyla* *nodiflora* (Linn.) Greene | Herb | Native |
| 109 | 鬼针草 | *Bidens* *pilosa* Linn. | Herb | Native |
| 110 | 狗牙根 | *Cynodon* *dactylon* (Linn.) Pers. | Herb | Alien |
| 111 | 狗尾草 | *Setaria* *viridis* (Linn.) Beauv. | Herb | Native |
| 112 | 附地菜 | *Trigonotis* *peduncularis* (Trev.) Benth. ex Baker et Moore | Herb | Native |
| 113 | 拂子茅 | *Calamagrostis* *epigejos* (L.) Roth, 1788 | Herb | Native |
| 114 | 饭包草 | *Commelina* *benghalensis* L. | Herb | Native |
| 115 | 萼距花 | *Cuphea* *hookeriana* Walp. | Herb | Alien |
| 116 | 鹅绒藤 | *Cynanchum* *chinense* R. Br. | Herb | Native |
| 117 | 钝叶酸模 | *Rumex* *obtusifolius* L. | Herb | Native |
| 118 | 钝叶决明 | *Senna* *obtusifolia* (L.) H. S. Irwin & Barneby, 1982 | Herb | Alien |
| 119 | 地锦草 | *Euphorbia* *humifusa* Willd. ex Schlecht. | Herb | Native |
| 120 | 地锦 | *Parthenocissus* *tricuspidata* (Siebold & Zucc.) Planch., 1887 | Herb | Native |
| 121 | 地黄 | *Rehmannia* *glutinosa* (Gaertn.) DC. | Herb | Native |
| 122 | 地肤 | *Bassia* *scoparia* (L.) A. J. Scott, 1978 | Herb | Alien |
| 123 | 打碗花 | *Calystegia* *hederacea* Wall. ex Roxb. | Herb | Native |
| 124 | 刺儿菜 | *Cirsium* *arvense* var. *integrifolium* C. Wimm. et Grabowski | Herb | Native |
| 125 | 垂序商陆 | *Phytolacca* *americana* Linn. | Herb | Alien |
| 126 | 垂盆草 | *Sedum* *sarmentosum* Bunge | Herb | Alien |
| 127 | 翅果菊 | *Lactuca* *indica* L. | Herb | Native |
| 128 | 车前草 | *Plantago* *asiatica* Ledeb. | Herb | Native |
| 129 | 朝天委陵菜 | *Potentilla* *supina* Linn. | Herb | Native |
| 130 | 彩叶草 | *Plectranthus* *scutellarioides* (L.) R. Br. | Herb | Alien |
| 131 | 北美苋 | *Amaranthus* *blitoides* S. Watson, 1877 | Herb | Alien |
| 132 | 半夏 | *Pinellia* *ternata* (Thunb.) Makino, 1901 | Herb | Alien |
| 133 | 斑地锦 | *Euphorbia* *maculata* L. | Herb | Alien |
| 134 | 稗 | *Echinochloa* *crus-galli* (L.) P. Beauv., 1812 | Herb | Alien |
| 135 | 白刺苋 | *Amaranthus* *spinosus* L., 1753 | Herb | Native |
| 136 | 毛白前 | *Vincetoxicum* *chinense* S. Moore, 1875 | Herb | Alien |
| 137 | 芭蕉 | *Musa* *basjoo* Siebold | Herb | Alien |
| 138 | 凹头苋 | *Amaranthus* *blitum* L., 1753 | Herb | Native |
| 139 | 艾草 | *Artemisia* *princeps* var. *orientalis* (Pamp.) Hara | Herb | Alien |
| 140 | 矮牵牛 | *Petunia* × *atkinsiana* (Sweet) D.Don ex W.H.Baxter | Herb | Alien |
